# Rheumatoid Arthritis With Multiple Lung Nodules: A Case Report

**DOI:** 10.7759/cureus.52350

**Published:** 2024-01-16

**Authors:** Mohammad A Alhajery

**Affiliations:** 1 Department of Internal Medicine, College of Medicine, Imam Mohammad Ibn Saud Islamic University (IMSIU), Riyadh, SAU

**Keywords:** laboratory evaluation, ct-guided biopsy, lungs, nodule, rheumatoid arthritis

## Abstract

Rheumatoid arthritis (RA) is an inflammatory multisystemic disease characterized by erosive arthritis with many extra-articular manifestations. Pleuropulmonary manifestations are frequently seen in patients with RA. Risk factors include male gender, severe erosive arthritis, high titers of rheumatoid factor, subcutaneous nodules, smoking, genetic predisposition, and the presence of other extra-articular manifestations of RA. We report a patient known to have RA presenting with multiple lung nodules. A 35-year-old female patient, known to have seropositive RA, was diagnosed 10 years ago. She was on oral corticosteroids (OCS) 5 mg daily, Upadacitinib 15 mg daily, and methotrexate (MTX) 20 mg weekly. The patient was referred for pulmonary medicine evaluation because of the finding of multiple lung nodules on chest imaging. A routine chest X-ray conducted as a part of the general evaluation showed a nodular opacity in the right lower lobe. Subsequently, a high-resolution CT (HRCT) scan of the chest was carried out and showed multiple pulmonary nodules. At the time of evaluation, she had no active respiratory symptoms with no signs of respiratory distress. As she was an active smoker, the decision was to proceed with a CT-guided biopsy besides full clinical, hematological, biochemical, and microbiological evaluations. The histopathological findings suggested a rheumatoid nodule with no evidence of malignant or infectious causes. No specific therapy was added at the time being, and the patient was monitored with regular follow-ups. Differentiation of rheumatoid lung nodules from other causes, such as malignancy and infectious causes, is essential. A biopsy with histopathological evaluation is a must in those with a high likelihood of malignancy, such as smokers. In addition to that, comprehensive clinical, hematological, microbiological, and radiological evaluations are required. Rheumatoid lung nodules are usually asymptomatic, with no specific therapy needed apart from the general management of RA with glucocorticoid, immunosuppressive, and biologic therapies.

## Introduction

Rheumatoid arthritis (RA) is an autoimmune disorder with an unidentified etiology that primarily impacts the joints. It is characterized by symmetrical polyarthritis with destruction and deformities of the joints [1,2]. Extraarticular manifestations are common in RA and are usually associated with more severe and typically seropositive disease [3]. Respiratory involvement is a common extra-articular manifestation of RA, with complications involving the pleura, pulmonary vascular system, airway, and lung parenchyma [4].

It can be manifested as upper airway disease, pleural effusion, interstitial lung disease (ILD), bronchiectasis, bronchiolitis obliterans, fibrosing alveolitis, Caplan syndrome, pulmonary rheumatoid nodules, pulmonary hemorrhage, organizing pneumonia, vasculitis, and pulmonary infections [5]. Among these, ILDs are the most common pulmonary manifestation of RA [5].

Risk factors of lung involvement in RA include male gender, middle age, severe erosive arthritis, high titers of rheumatoid factor (RF), subcutaneous nodules, smoking, genetic predisposition, and presence of other extra-articular manifestations of RA [6].

Extra Ellman and Ball were the first to describe pulmonary involvement in RA when they noted diffuse pulmonary fibrosis in three patients with the disease [3]. Up to 32% of individuals with RA have been diagnosed with rheumatoid lung nodules; a more detailed evaluation is required to exclude neoplasms, tuberculosis (TB), and fungal infection [4].

The associated morbidity and severity increase and the life expectancy can be reduced by 5 to 10 years when RA involves other organs besides the joints [2]. We report a patient known to have seropositive RA, presenting with multiple lung nodules.

## Case presentation

A 35-year-old female patient, known to have been diagnosed with seropositive RA 10 years ago, was maintained on MTX. In the past, she experienced frequent articular flare-ups of her disease, necessitating management with repeated courses of oral corticosteroids (OCSs).

For the last three years, she was noncompliant with RA treatment and did not attend follow-ups with her rheumatologist. She sought medical advice again in early 2023 due to severe joint pain. Since her RA was active and uncontrolled, her rheumatologist initiated treatment with OCSs, gradually tapering to 5 mg daily, Upadacitinib 15 mg daily, and MTX 20 mg per week. She was referred to pulmonary medicine because of the incidental finding of pulmonary nodules discovered on a routine chest X-ray that was followed by a high-resolution computed tomography (CT) scan of the chest.

History

Upon clinical evaluation, she had no active respiratory symptoms. There was no significant cough, shortness of breath, or respiratory functional limitation. She had no history of hemoptysis, fever, night sweats, loss of weight, or appetite. She denied a history of TB or contact with a known TB case, recent travel, or a previous history of malignancy. There was no family history of lung cancer, malignancy, or history of chronic lung diseases. She worked in an office-based job and had no occupational or animal exposure. She was an active smoker, smoking Shisha (Hookah) three to four times per week for the last five years. 

Observational evaluation

She was fully conscious, alert, and oriented. Vital signs were within normal limits (SPO_2_, 99% on room air; blood pressure, 118/78 mmHg; temperature, 36.8 °C; pulse rate, 70 beats/minute; respiratory rate, 18 breaths/minute), and no signs of respiratory distress were noted. During the observational evaluation, no finger clubbing or subcutaneous nodules were noted. The patient's chest evaluation showed bilateral vesicular breathing with no added sounds. Cardiovascular evaluation was unremarkable, with no signs of pulmonary hypertension observed. 

Radiological evaluation

**Figure 1:**
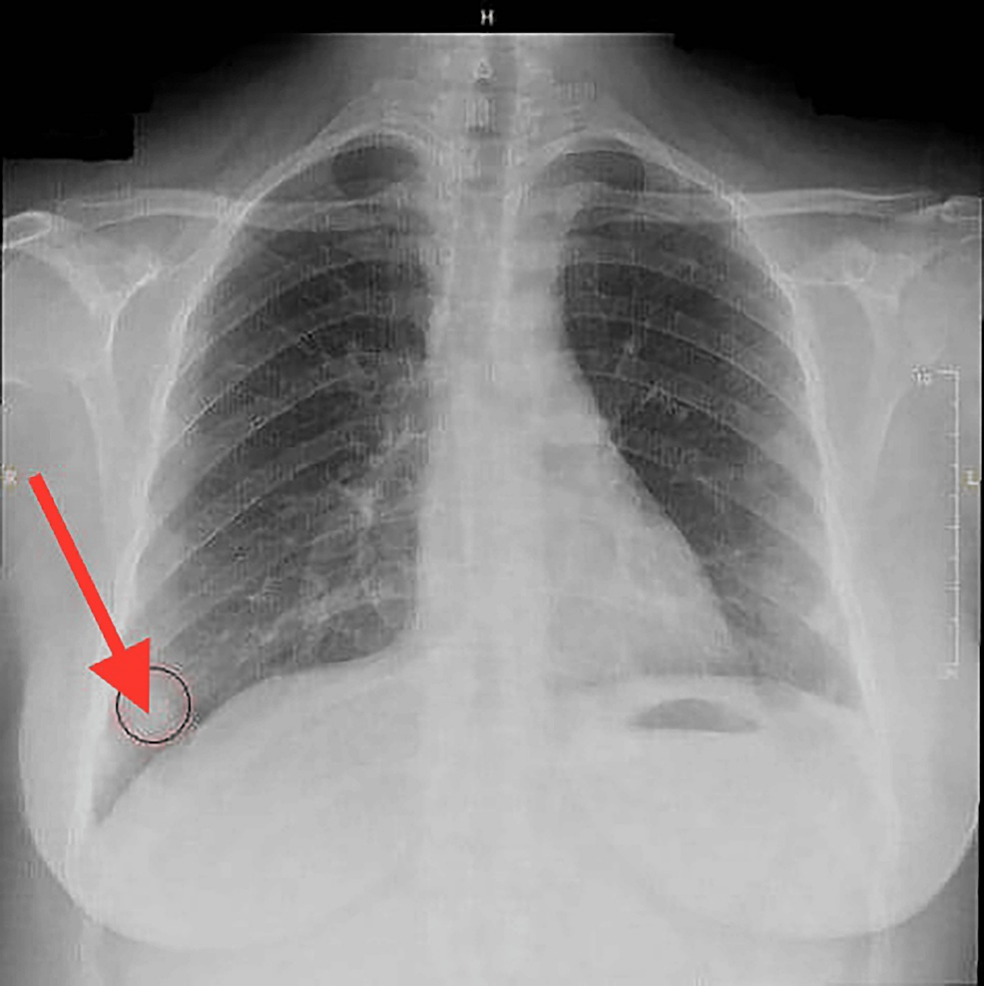
The chest X-ray of the patient showing a nodular opacity in the right lower lobe (July 22, 2023).

As part of the general evaluation, a routine chest X-ray was performed, revealing a nodular opacity in the right lower lobe (Figure 1). 

**Figure 2:**
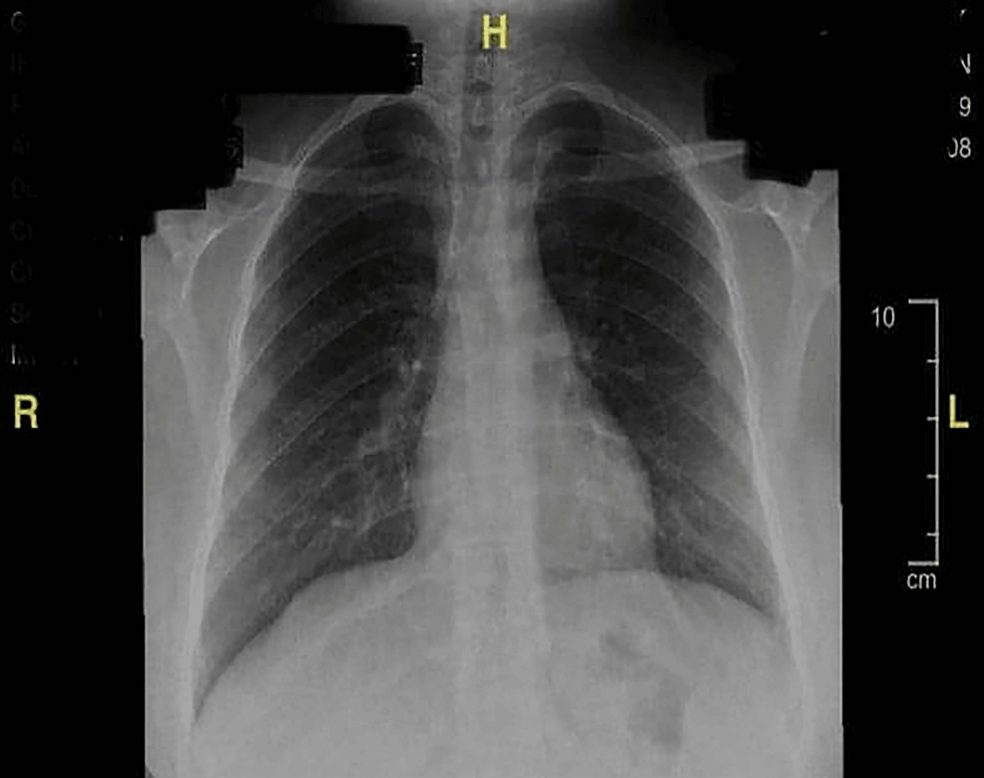
Chest X-ray of the patient conducted in October 18, 2019.

Of note, the previous chest X-ray conducted in 2019 (Figure 2) was unremarkable compared to the most recent one.

**Figure 3:**
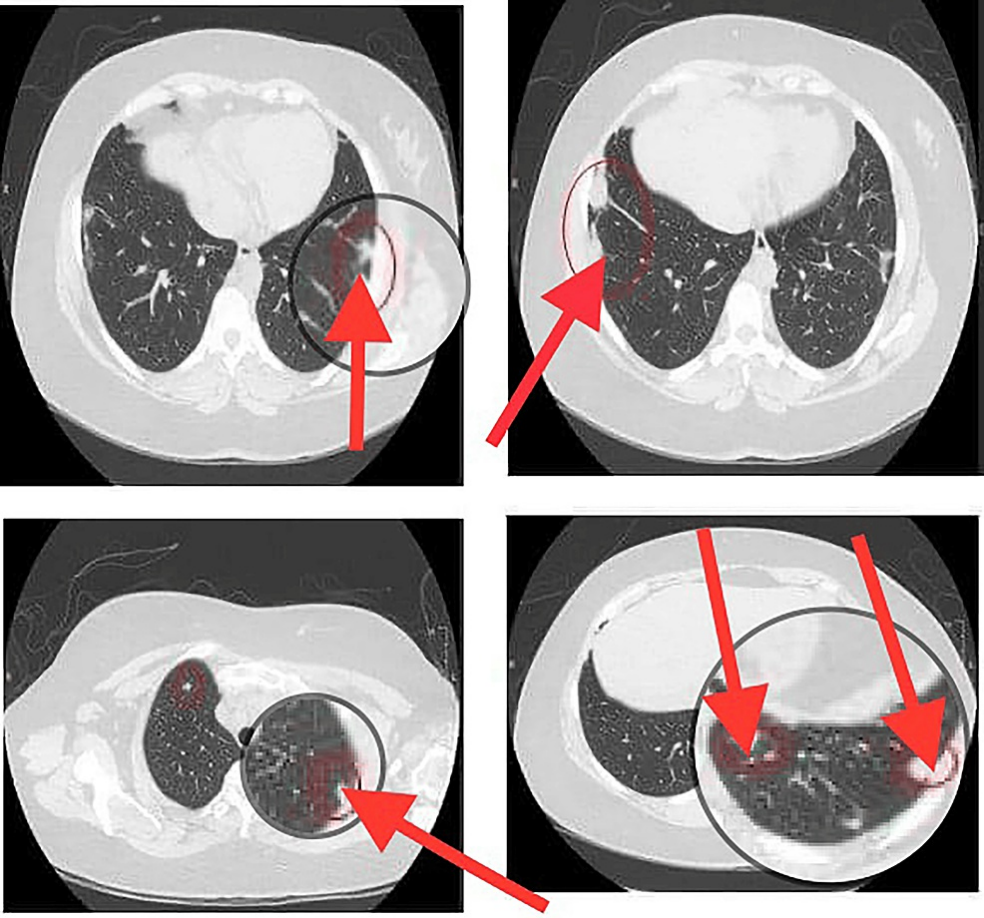
Computed tomography (CT) of the thorax revealing multiple variably sized, ill-defined pulmonary lesions/nodules at the periphery of both lower lobes, along with small nodules observed at the periphery of both upper lobes (July 23, 2023).

Following that, a chest high-resolution CT (HRCT) scan was performed, which showed multiple variables in size and ill-defined pulmonary lesions/nodules at the periphery of both lower lobes, the largest one measuring 3 cm x 1.4 cm. Small nodules were seen at the periphery of both upper lobes. No airspace consolidation, masses, cavitary lung lesions, interlobular septal thickening, or honeycombing were observed. Multiple subcentimeter lymph nodes were seen in the mediastinum. The major vascular structures were grossly unremarkable; major airways were patent, and no pericardial effusion, pleural effusion, pneumothorax, or significant abnormalities in visualized bone were observed (Figure 3). 

Laboratory evaluation

Laboratory examination revealed normal liver and renal function tests. The patient's RF was 229.5 U/mL, and anti-cyclic citrullinated peptide (anti-CCP) was 13.60 U/mL. The complete blood count showed a low level of hemoglobin (10.7 gm/dL) and altered red cell indices concentration. The total leukocyte count (13,460 cu/mm), platelet count (5.07 lakh cu/mm), and ESR level (63 mm/hour) were high in the patient.

Further evaluation with CT-guided biopsy

Based on initial clinical, laboratory, and radiological evaluation, rheumatoid nodules were likely the cause. Nonetheless, other causes, including malignancy and infectious etiologies, cannot be ruled out, particularly given her history of smoking and current use of immunosuppressive medications.

**Figure 4:**
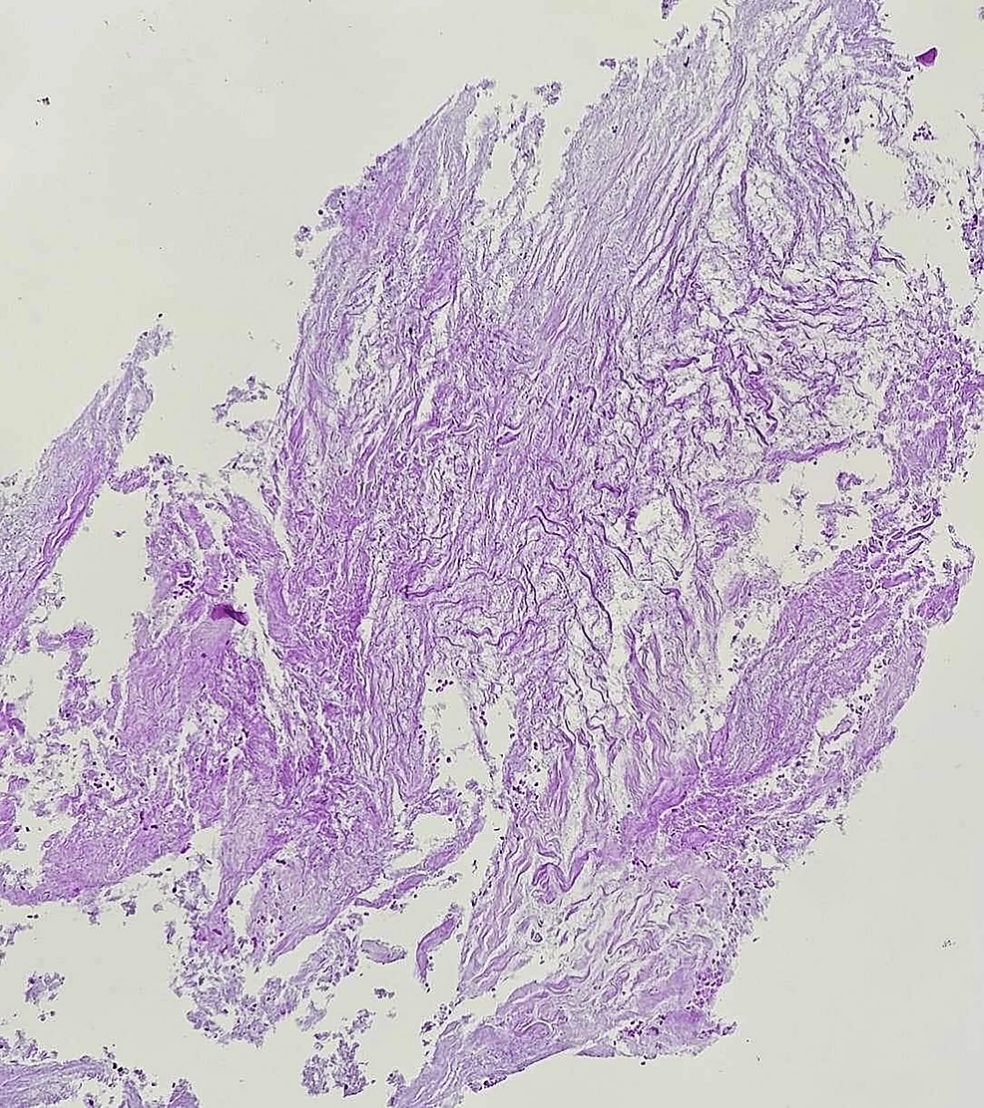
CT-guided biopsy of the nodule (August 10, 2023). CT, computed tomography

After a full discussion with the patient, a CT-guided biopsy of the largest accessible nodule was carried out. The biopsy specimen was kept in formalin and sent for cytology and histopathology. The histopathological evaluation showed benign loose connective tissue with foci of degenerative changes and was negative for malignancy. The histological investigation indicated necrotizing inflammation with clusters of macrophages, lymphocytes, and plasma cells surrounding the necrotic area. There was no evidence of vasculitis or cancer. No granuloma was observed. Microbiological examinations were negative, including bacterial, mycobacterial, and fungal cultures (Figure 4).

Final diagnosis and plan

The patient was diagnosed with rheumatoid lung nodules. No specific therapy was added to the general management of RA with glucocorticoid, immunosuppressive, and biologic therapies, which were already started for the patient by our colleagues in Rheumatology Medicine. The patient was strongly advised to quit smoking and to be compliant with RA treatment. The patient will be monitored closely for respiratory symptoms, with regular follow-ups and clinical evaluation. Further workup will be done if clinically indicated.

## Discussion

Lung nodules are one of the extra-articular manifestations in patients with RA [1]. The prevalence of these nodules varies: only 1% of chest X-rays in patients with RA detect them, whereas 20% to 22% are detected by HRCT [7].

Males and smokers with concomitant subcutaneous nodules and high titers of RF are most commonly affected by rheumatoid pulmonary nodules [8]. The lesion usually does not show symptoms. As described in this and the previous cases, it is associated with seropositive cases (positive RF) and MTX treatment [9]. An interruption to MTX medication decreases the size of rheumatoid nodules, as reported repeatedly in different studies [10].

The presence of lung nodules in patients with RA is a diagnostic challenge. It is important to rule out the possibility of malignancy and infectious causes, such as TB [11]. Nodules with irregular borders and diameters exceeding 10 mm are a cause for concern regarding lung cancer. Metastasis can also appear as multiple lung nodules and should always be considered a possible cause [12]. A biopsy is a must if there is a high likelihood of malignancy. In a patient with a classic history of RA who does not smoke and has no clinical or imaging features of concern, a biopsy of a lung nodule may not be necessary. However, a close follow-up is required with a low threshold to proceed with biopsy if clinically indicated. 

In this case, the patient's significant smoking history and the presence of large-sized nodules were crucial factors in the decision to proceed with the biopsy.

Most rheumatoid lung nodules are asymptomatic with no recommended specific therapy and usually remit spontaneously without any relation to the evolution of arthritis [9].

## Conclusions

Lung nodules are one of the extra-articular manifestations seen in patients with RA. Differential diagnosis is wide, including malignant and infectious causes such as TB. Detailed clinical, hematological, biochemical, microbiological, and radiological evaluations are needed. A biopsy with histopathological evaluation is essential in individuals with a high likelihood of malignancy, such as smokers. Rheumatoid lung nodules are usually asymptomatic and do not require a specific therapy. Close monitoring and follow-ups with additional investigations as clinically indicated are recommended.
